# Incidence of and Risk Factors for Subsequent Lower Respiratory Tract Infection Following an Infant RSV Hospitalization

**DOI:** 10.3390/children12020183

**Published:** 2025-02-02

**Authors:** Rees Lee, Tan Ding, Corinne A. Riddell, Tina Hartert, Pingsheng Wu

**Affiliations:** 1 Department of Pediatrics, College of Medicine, University of Arizona, Tucson, AZ 85721, USA; reeslee@arizona.edu; 2 Department of Biostatistics, Vanderbilt University Medical Center, Nashville, TN 37203, USA; tan.ding@vumc.org; 3 Divisions of Biostatistics and Epidemiology, School of Public Health, University of California, Berkeley, CA 94720, USA; c.riddell@berkeley.edu; 4 Department of Medicine, Vanderbilt University Medical Center, Nashville, TN 37203, USA; tina.hartert@vumc.org; 5 Department of Pediatrics, Vanderbilt University Medical Center, Nashville, TN 37203, USA

**Keywords:** respiratory syncytial virus, medically attended lower respiratory tract infection, bronchiolitis, infant

## Abstract

**Background/Objectives**: Respiratory syncytial virus (RSV) is the most common cause of bronchiolitis and pneumonia in infants and the leading cause of infant hospitalization in the U.S. and worldwide. The risk of experiencing at least one other medically attended lower respiratory tract infection (MA LRTI) following an infant RSV hospitalization is less studied. **Methods**: We conducted a retrospective cohort study of infants who experienced an RSV hospitalization (index hospitalization) during infancy. The incidence rate of having a subsequent MA LRTI was reported. The association between a priori selected maternal and infant risk factors and subsequent MA LRTI was determined. **Results**: Of the 20,181 children who experienced an RSV hospitalization in infancy, 15% had at least one subsequent MA LRTI within the same RSV season. The incidence rates (95% confidence interval) of having a subsequent MA LRTI hospitalization, emergency department visit, or physician office visit in the same RSV season were 0.27 (0.26, 0.29), 0.16 (0.15, 0.17), and 0.46 (0.44, 0.48) per infant-year, respectively. Factors associated with an increased risk of subsequent MA-LRTI include younger maternal age, fewer years of maternal education, smoking during pregnancy, cesarean delivery, male infant sex, White race, siblings at home, urban residence, lower birth weight, lower gestational age, eligibility for and/or ever receiving palivizumab, longer birth hospitalization length of stay, longer index RSV hospitalization length of stay, intensive care unit admission for the index hospitalization, and summer-to-fall births. **Conclusions**: The burden of clinically significant subsequent MA-LRTI following an RSV hospitalization can be substantial. Our results highlight the importance of increasing accessible RSV LRTI preventive interventions.

## 1. Introduction

Respiratory syncytial virus (RSV) is the most common cause of bronchiolitis and pneumonia in infants and the leading cause of infant hospitalization in the U.S. and respiratory mortality worldwide [1]. RSV immunoprophylaxis, limited to palivizumab until 2023, is an effective strategy in reducing morbidity [2]. The risk of having two RSV hospitalizations in the same season is thought to be extremely low (<0.5%), prompting the current American Academy of Pediatrics (AAP) to advise discontinuation of palivizumab following the first breakthrough RSV hospitalization [2]. This recommendation is different from what the Advisory Committee on Immunization Practices recommends for the extended half-life monoclonal antibody nirsevimab, which they recommend regardless of prior RSV infections or RSV-associated hospitalization. The objective of this study was to determine an infant’s risk of experiencing at least one other medically attended lower respiratory tract infection (MA-LRTI) that required an unscheduled healthcare visit following an initial RSV LRTI hospitalization in the same RSV season. Results of this study will elucidate the burden of and risk factors for subsequent MA-LRTIs in infancy.

## 2. Materials and Methods

**Figure 1:**
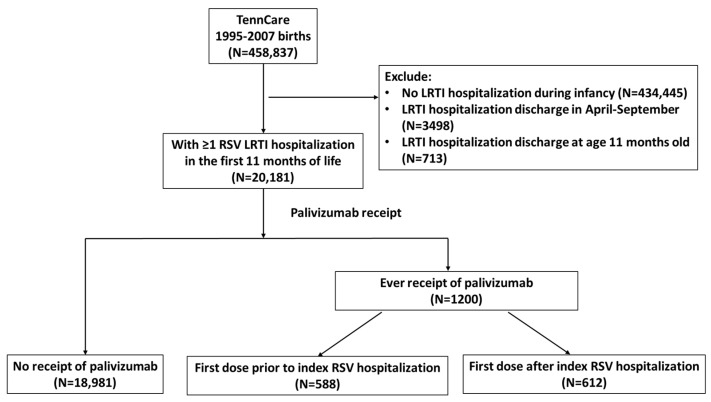
Flow diagram of the study population (N = 20,181).

We conducted a retrospective cohort study of infants born between 1995 and 2007, who were continuously enrolled in the Tennessee Medicaid Program (TennCare) and experienced an RSV LRTI hospitalization (index hospitalization) during the winter virus season in the northern hemisphere region (October–March) of the first eleven months of life (N= 20,181) (Figure 1). The RSV hospitalization was defined using a validated algorithm using International Classification of Diseases, Ninth Revision (ICD-9) codes for acute bronchiolitis (466.1) and/or RSV pneumonia (480.1) [3]. We limited the index hospitalization to the first eleven months of life to allow at least one day of follow-up time during infancy, which started 30 days after the index hospitalization discharge. Follow-up time for each child began 30 days after discharge from the index RSV LRTI hospitalization and ended at the admission date of the subsequent most severe MA-LRTI event (if they had one), April 30 of the RSV season in which the index hospitalization occurred, or the child’s first birthday, whichever occurred first (Figure S1). The subsequent MA-LRTI was determined using the same algorithm for the index RSV LRTI hospitalization with severity defined as no visit documented < physician office visit < emergency department (ED) visit < hospitalization. The incidence rates and their 95% confidence intervals (CI) of having a subsequent MA-LRTI per healthcare type were reported. We a priori selected a set of maternal and infant factors and performed univariate Cox proportional hazard models to estimate which risk factors were associated with increased risk of having a subsequent MA-LRTI. We did not perform an adjusted analysis to avoid effect cancelation among correlated variables. Proportional hazard assumptions were evaluated and met. We further reported incidence rates separately for children who never received palivizumab, first dose receipt prior to the index RSV hospitalization, and first dose receipt after the index RSV hospitalization. All analyses were performed using the R software version 4.3.2 [4]. This study was a secondary data analysis for which individual consent was not required. We had no access to information that could identify individual participants during or after data collection. The study protocol was approved by Vanderbilt University Medical Center and the Tennessee Department of Health Institutional Review Board.

## 3. Results

There were 20,181 infants with an index RSV LRTI hospitalization during their first eleven months of life. They were predominantly male (57.3%), White (73.2%), and born at term (80.8%). The median age (interquartile range [IQR]) at the index RSV hospitalization was 3.9 (2.0–6.4) months (Table 1).

During a total of 3273.6 infant-years of follow-up time, 3035 (15.0%) infants developed at least one additional MA-LRTI requiring another hospitalization (4.9%), emergency department visit (2.8%), or a physician office visit (7.4%). The incidence rates (95% CI) of having a subsequent LRTI hospitalization, ED visit, or physician office visit in the same RSV season of infancy were 0.27 (0.26, 0.29), 0.16 (0.15, 0.17), and 0.46 (0.44, 0.48) per infant-year, respectively. Children of younger mothers had a higher hazard of having a subsequent MA-LRTI. Lower maternal education, smoking during pregnancy, and cesarean delivery were associated with an increased hazard of subsequent MA-LRTI (Table 1). Children with a subsequent MA-LRTI were more likely to be male, White, with older siblings, living in an urban residence, lower in birth weight, lower in gestational age, with chronic lung disease, eligible for and/or ever received palivizumab, and have longer birth hospitalization length of stay. Infants born in January-March had the lowest risk of having a subsequent MA-LRTI, while children born in July through September had the highest risk. Children with a subsequent MA-LRTI were also more likely to have the index RSV hospitalization during the first 6 months of life vs. 7–11 months, with a longer index hospitalization length of stay and a higher likelihood to be admitted to the intensive care unit (Table 1).

Twelve hundred (5.9%) infants received at least one dose of palivizumab. The incidence rates (95% CI) of having a subsequent MA-LRTI hospitalization, ED visit, or physician office visit were 0.26 (0.24, 0.27), 0.15 (0.14, 0.17), and 0.46 (0.43, 0.48) per infant-year for infants who never received palivizumab, 0.54 (95%CI: 0.42, 0.71), 0.22 (95% CI: 0.14, 0.33), and 0.42 (95% CI: 0.31, 0.58) for infants who had their first dose of palivizumab prior to the index hospitalization and 0.58 (95% CI: 0.46, 0.74), 0.29 (95% CI: 0.21, 0.42), and 0.52 (95% CI: 0.40, 0.69) for infants whose first dose was given after the index hospitalization, respectively.

## 4. Discussion

In this retrospective cohort study of infants who experienced an RSV hospitalization, 15% had at least one subsequent MA-LRTI within the same RSV season. The incidence rate of having subsequent MA-LRTI was even higher in infants who ever received palivizumab, often an indication of increased risk for RSV LRTI. While having an RSV hospitalization likely provides some natural immunity and may decrease the risk of having subsequent RSV LRTIs, our finding suggests that a second significant LRTI is more likely in infants than previously appreciated [2]. AAP guidelines for palivizumab, based on small studies that included children up to 5 years of age, recommend the discontinuation following a breakthrough RSV hospitalization [2]. The risk of RSV and RSV reinfection is highest in infancy and declines rapidly with age such that including older children in studies may mask the real risk to those under 1 year of age [5]. In a Canadian database study of children with an initial RSV hospitalization < 5 years of age, the risk of RSV-related rehospitalization was reported to be 0.35%, ten times smaller than the overall LRTI rehospitalization rate for < 1 year of age reported in this study (4.9%) [6]. Although the higher LRTI rehospitalization rate observed cannot be attributed specifically to RSV, given that the majority of severe LRTIs infants encounter during the RSV season and during the first year of life are likely RSV-related [7], recurrent RSV infection cannot be excluded, and further research of RSV-specific subsequent LRTIs is warranted. Further, the economic burden of having another MA-LRTI could be substantial. Using US MarketScan data, Tran and colleagues reported medical costs of $28,586 (standard deviation: 55,523) for an MA RSV LRTI episode that included an inpatient stay and $2099 (standard deviation: 12,829) for an outpatient-only MA RSV LRTI episode for infants less than one year of age [8]. With an average of 2.6 days of caretaker absenteeism while the child is sick [9,10], the societal and economic burden could be even higher. The potential risk of RSV following an RSV LRTI and the associated economic burden should be considered in determining when discontinuation of RSV immunoprophylaxis is most appropriate.

We acknowledge the limitations of this study. We could not confirm RSV infection by laboratory testing, although the algorithm we used has been validated previously based on viral identification and is consistent for the index hospitalization and the subsequent MA-LRTI [3]. The study included children born 1995–2007; however, the risk factors for another MA-LRTI and clinical criteria for LRTI hospitalization have not changed substantially in the past 20 years. Given that RSV LRTI in infants and young children remains a significant public health issue and the increasing availability of nirsevimab and maternal RSV vaccine in the US and worldwide, we believe findings based on this study population are still relevant [11,12]. Nevertheless, studies with more recent data and with LRTIs with known etiology are needed to inform policy. Our study population includes subjects who qualify for Medicaid either based on income or significant chronic illness. While this represents more than 50% of one state’s annual births, the results from our study may not be generalizable to other populations.

## 5. Conclusions

In conclusion, we reported that 15% of infants experience at least one other MA-LRTI after an RSV hospitalization in the same season, and approximately half of them were severe enough to require hospitalization or an ED visit. Younger maternal age, lower maternal education, smoking during pregnancy, cesarean delivery, male infant sex, White race, having older siblings, urban residence, lower birth weight, lower gestational age, chronic lung disease, eligibility for and/or ever receipt of palivizumab, longer birth hospitalization length of stay, longer index hospitalization length of stay, intensive care unit admission for the index hospitalization, and summer-to-fall births were associated with an increased risk of having a second MA-LRTI in the same season of infancy. Our findings provide new evidence on the burden of clinically significant subsequent LRTIs following an RSV hospitalization during infancy. With maternal vaccines and extended half-life monoclonal antibodies increasingly available to pregnant women and all infants, respectively, further research using more recent data on RSV-specific reinfection and readmission rates together with laboratory confirmation of the viral agents is needed to inform policy recommendations.

## Figures and Tables

**Table 1:** Maternal and infant demographic characteristics of children who experience an RSV LRTI hospitalization during the winter RSV season (October—March) of the first eleven months of life (N = 20,181) and the hazard ratio (HR) for the occurrence of a subsequent MA-LRTI during the same RSV season.

Characteristic	n (%)	HR (95% CI)
Maternal age at delivery (years) (N = 20,145)	22 (20–26) ^1^	
<18	1712 (8.5%)	1.04 (0.91, 1.18)
18–24	12,047 (59.8%)	reference
25–29	4194 (20.8%)	0.95 (0.86, 1.04)
30–34	1545 (7.7%)	0.94 (0.82, 1.08)
≥35	647 (3.2%)	0.68 (0.54, 0.86)
Maternal smoking during pregnancy (N = 20,145)	7312 (36.3%)	1.10 (1.02, 1.19)
Maternal education (years) (N = 20,131)		
<12	8410 (41.8%)	1.04 (0.96, 1.12)
12	8761 (43.5%)	reference
>12	2960 (14.7%)	0.85 (0.76, 0.95)
Mode of delivery (N = 20,172)		
Vaginal	13,013 (64.5%)	reference
Assisted	1539 (7.6%)	1.06 (0.93, 1.21)
C-section	5620 (27.9%)	1.14 (1.05, 1.23)
Infant male sex	11,568 (57.3%)	1.25 (1.16, 1.34)
Infant race		
White	14,766 (73.2%)	reference
African American	5173 (25.6%)	0.91 (0.83, 0.98)
Other	242 (1.2%)	0.63 (0.43, 0.92)
Number of older siblings at home (N = 20,148)	1 (0, 2) ^1^	
0	6856 (34.0%)	reference
1	6749 (33.5%)	1.18 (1.08, 1.29)
≥2	6543 (32.5%)	1.16 (1.06, 1.26)
Residence (N = 20,156)		
Urban	9602 (47.6%)	reference
Standard Metropolitan Statistical Area	4960 (24.6%)	0.78 (0.72, 0.86)
Rural	5594 (27.8%)	0.70 (0.64, 0.76)
Birth weight (grams)	3118 (2722–3487) ^1^	
<1500	749 (3.7%)	1.43 (1.22, 1.69)
1500–<2500	2593 (12.8%)	1.22 (1.11, 1.35)
2500–<4000	15,838 (78.5%)	reference
≥4000	1001 (5.0%)	0.92 (0.77, 1.10)
Gestational age (weeks)	39.1 (37.1–40.1) ^1^	
<29	405 (2.0%)	1.58 (1.28, 1.94)
29–32	746 (3.7%)	1.22 (1.02, 1.45)
33–36	2722 (13.5%)	1.18 (1.07, 1.30)
≥37	16,308 (80.8%)	reference
Birth hospitalization length of stay (days)	6 (3–7) ^1^	
≤3	5629 (27.9%)	reference
4–7	10,129 (50.2%)	0.91 (0.84, 0.99)
8–30	3450 (17.1%)	1.10 (0.99, 1.22)
≥31	973 (4.8%)	1.24 (1.06, 1.45)
Birth month		
January–March	3216 (15.9%)	reference
April–June	3619 (17.9%)	1.11 (0.96, 1.28)
July–September	6189 (30.7%)	1.24 (1.09, 1.42)
October–December	7157 (35.5%)	1.05 (0.92, 1.20)
Chronic lung disease	201 (1.0%)	1.50 (1.11, 2.01)
Congenital heart disease	196 (1.0%)	1.15 (0.82, 1.61)
Eligible for receipt of palivizumab ^2^	877 (4.3%)	1.50 (1.30, 1.73)
Receipt of palivizumab		
Never	18,981 (94.1%)	reference
First dose receipt prior to index hospitalization	588 (2.9%)	1.41 (1.17, 1.70)
First dose receipt after index hospitalization	612 (3.0%)	1.73 (1.47, 2.03)
Age at the index RSV hospitalization (months)	3.9 (2.0–6.4) ^1^	
≤2	4996 (24.8%)	1.23 (1.11, 1.37)
3–6	9425 (46.7%)	1.41 (1.29, 1.55)
7–11	5760 (28.5%)	reference
Length of stay of the index RSV hospitalization (days)	4 (3–5) ^1^	
1–2	3851 (19.1%)	reference
3–7	14,430 (71.5%)	1.05 (0.95, 1.15)
≥8	1900 (9.4%)	1.32 (1.15, 1.51)
Intensive care unit admission during the index RSV LRTI hospitalization	853 (4.2%)	1.29 (1.10, 1.51)

^1^ Median (interquartile range). ^2^ Eligibility determined based on American Academy of Pediatrics guidelines specific for infants’ birth year. RSV: respiratory syncytial virus; LRTI: lower respiratory tract infection; HR: hazard ratio.

## Data Availability

The data to support the findings of this study were from the Division of TennCare in the Tennessee Department of Finance and Administration. De-identified data are available on request from the corresponding author, with the approvals from TennCare and Vanderbilt University Medical Center Institutional Review Board. Data are not publicly available due to privacy concerns and regulatory compliance.

## Supplementary Materials

The following supporting information can be downloaded at https://www.mdpi.com/article/10.3390/children12020183/s1: Figure S1: Data processing strategy depicting hypothetical infants who were followed from 30 days after the index RSV hospitalization discharge to the admission date of the subsequent most severe MA-LRTI event (if they had one), April 30 of the RSV season in which the index hospitalization occurred, or the child’s first birthday, whichever occurred first. Infants #1 and #2 present cases whose 1st birthday falls outside the RSV season, while infants #3 and #4 have birthdates within the RSV season.

## Abbreviations

The following abbreviations are used in this manuscript:

AAP	American Academy of Pediatrics
CI	Confidence interval
ED	Emergency department
HR	Hazard ratio
ICD-9	International Classification of Diseases, Ninth Revision
LRTI	lower respiratory tract infection
MA	Medically attended
RSV	Respiratory syncytial virus
TennCare	Tennessee Medicaid Program
