# Supplementary material for: Incidence of and Risk Factors for Subsequent Lower Respiratory Tract Infection Following an Infant RSV Hospitalization
Source: Children (Basel). 2025 Feb 2;12(2):183. doi: 10.3390/children12020183 (PMC11854575; doi:10.3390/children12020183)

Subsequent lower respiratory tract infection following an infant RSV hospitalization

Rees Lee; Tan Ding, Corinne Riddell, Tina Hartert, Pingsheng Wu

Supplemental Figure S1. Data processing strategy depicting hypothetical infants who were followed from 30 days after the index RSV hospitalization discharge to the admission date of the subsequent most severe MA-LRTI event (if they had one), April 30 of the RSV season in which the index hospitalization occurred, or the child's first birthday, whichever occurred first. Infants #1 and #2 present cases whose 1st birthday falls outside the RSV season while infants #3 and #4 have birthdates within the RSV season.

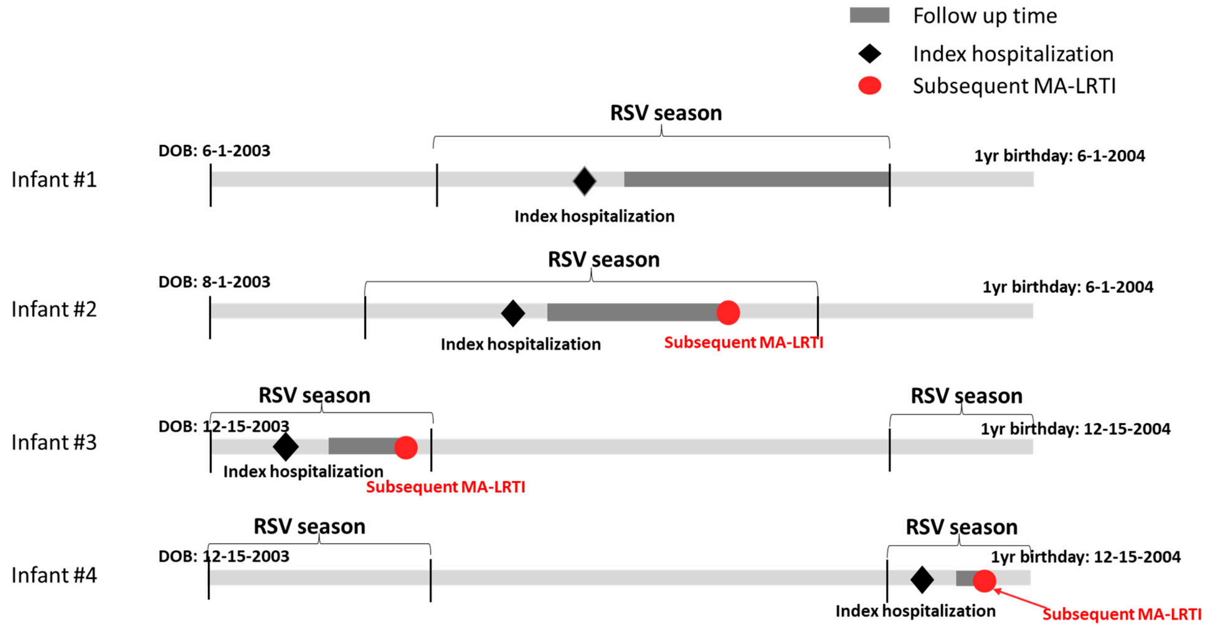

Supplement: Supplementary file 1 [file children-12-00183-s001.zip › children-3410450-supplementary.pdf]
